# Recurrent Die-Offs of Adult Coho Salmon Returning to Spawn in Puget Sound Lowland Urban Streams

**DOI:** 10.1371/journal.pone.0028013

**Published:** 2011-12-14

**Authors:** Nathaniel L. Scholz, Mark S. Myers, Sarah G. McCarthy, Jana S. Labenia, Jenifer K. McIntyre, Gina M. Ylitalo, Linda D. Rhodes, Cathy A. Laetz, Carla M. Stehr, Barbara L. French, Bill McMillan, Dean Wilson, Laura Reed, Katherine D. Lynch, Steve Damm, Jay W. Davis, Tracy K. Collier

**Affiliations:** 1 Northwest Fisheries Science Center, NOAA Fisheries, Seattle, Washington, United States of America; 2 Department of Natural Resources and Parks, King County, Seattle, Washington, United States of America; 3 Wild Fish Conservancy, Duvall, Washington, United States of America; 4 Seattle Public Utilities, City of Seattle, Seattle, Washington, United States of America; 5 Washington Fish and Wildlife Office, U.S. Fish and Wildlife Service, Lacey, Washington, United States of America; Institute of Marine Research, Norway

## Abstract

Several Seattle-area streams in Puget Sound were the focus of habitat restoration projects in the 1990s. Post-project effectiveness monitoring surveys revealed anomalous behaviors among adult coho salmon returning to spawn in restored reaches. These included erratic surface swimming, gaping, fin splaying, and loss of orientation and equilibrium. Affected fish died within hours, and female carcasses generally showed high rates (>90%) of egg retention. Beginning in the fall of 2002, systematic spawner surveys were conducted to 1) assess the severity of the adult die-offs, 2) compare spawner mortality in urban vs. non-urban streams, and 3) identify water quality and spawner condition factors that might be associated with the recurrent fish kills. The forensic investigation focused on conventional water quality parameters (e.g., dissolved oxygen, temperature, ammonia), fish condition, pathogen exposure and disease status, and exposures to metals, polycyclic aromatic hydrocarbons, and current use pesticides. Daily surveys of a representative urban stream (Longfellow Creek) from 2002–2009 revealed premature spawner mortality rates that ranged from 60–100% of each fall run. The comparable rate in a non-urban stream was <1% (Fortson Creek, surveyed in 2002). Conventional water quality, pesticide exposure, disease, and spawner condition showed no relationship to the syndrome. Coho salmon did show evidence of exposure to metals and petroleum hydrocarbons, both of which commonly originate from motor vehicles in urban landscapes. The weight of evidence suggests that freshwater-transitional coho are particularly vulnerable to an as-yet unidentified toxic contaminant (or contaminant mixture) in urban runoff. Stormwater may therefore place important constraints on efforts to conserve and recover coho populations in urban and urbanizing watersheds throughout the western United States.

## Introduction

In lowland Puget Sound, many urban streams in the vicinity of Seattle were a focus of extensive physical and biological restoration activities in the 1990s. These projects, sponsored by the City of Seattle and other regional municipalities, served multiple purposes such as the creation of public green space, the removal of culverts and other impassable barriers for fish, the placement of large woody debris and gravel substrate, the removal of noxious weeds, and the planting of native vegetation. A related aim was to evaluate the extent to which adult salmon would return to spawn in the newly available and improved habitats. This post-project effectiveness monitoring was carried out via fall spawner surveys that were conducted weekly from 1999–2001, with a primary focus on coho (*Oncorhynchus kisutch*), Chinook (*O. tshawytscha*) and chum (*O. keta*) salmon.

These early monitoring efforts in 1999–2001 identified an unusual syndrome of pre-spawn mortality among adult coho returning to restoration sites to spawn. Coho typically spawn in small lowland streams in October through December. Eggs incubate in gravel nests (redds) from which fry emerge in the spring (March through May). Juveniles rear in freshwater for approximately a year and then outmigrate to estuaries the following spring. Coho spend at least one full year in the ocean before returning to their natal watersheds to spawn, after which they die (semelparous life history). Adult migration into freshwater is triggered by fall rain events that produce transient high flows in streams. Coho spawning in Seattle-area streams are often a mix of hatchery and natural origins, with hatchery fish distinguishable by a clipped adipose fin and, less commonly, the presence of a rostral-implant coded wire tag.

Affected coho spawners observed in post-restoration effectiveness monitoring surveys showed a consistent suite of symptoms that included surface swimming, gaping, loss of equilibrium, and pectoral fin splaying (Video S1). The onset of the syndrome was rapid, and stricken fish typically died within a few hours. Pre-spawn mortality was confirmed by a near-total retention of eggs in female carcasses inspected during the surveys.

The recurrent die-off of coho in urban drainages appears to be a phenomenon distinct from other types of pre-spawn mortality that have previously been reported for other species of salmon. These include, for example, sockeye salmon in the Fraser River and watersheds of Bristol Bay, as well as Chinook salmon in the Klamath River. In those non-urban freshwater habitats, pre-spawn mortality is described as a chronic process where fish are weakened by a low energy status, poor physical condition, wasting, and eventual death. This process occurs over a protracted timeframe (i.e., weeks). The causes vary and include an abnormally early arrival on spawning grounds, thermal stress, and increased susceptibility to the myxosporean parasite *Parvicapsula minibicornis* (Fraser River sockeye; [1], [2], [3]); high spawner density, low water level, high water temperature, and low dissolved oxygen levels (Bristol Bay sockeye; [4], [5]); and low flows, increased water temperature, high spawner densities, and diseases caused by the pathogenic ciliate *Ichtyophthirius multifulis* and *Flavobacterium columnare*, the bacterial agent for columnaris in fish (e.g., Klamath River Chinook salmon; [6]).

Here we report the results of an eight-year investigation (2002–09) to characterize the frequency and geographical extent of coho mortality, and to identify associated water quality and spawner condition factors. We conducted daily surveys of multiple creeks to assess rates of pre-spawning mortality across the entire duration of fall coho runs. We assessed the physical condition, pathogen exposure status, and disease status of affected female coho for comparison to 1) unaffected wild adult females collected from a non-urban reference stream, 2) unaffected adult females returning to several area hatcheries, and 3) seawater-phase adults collected from Elliott Bay along the Seattle waterfront, prior to their entry to restored freshwater habitats in urban drainages. Fish from a subset of these locations were profiled using biomarkers of exposure to common toxic contaminants in urban runoff, including metals, homeowner use insecticides, and petroleum hydrocarbons. Lastly, we monitored conventional water quality information (e.g., temperature, dissolved oxygen, specific conductance, and pH) for urban streams during adult coho die-off events.

## Materials and Methods

A paucity of coho spawners in urban streams throughout this study placed important constraints on sample collection for the purposes of a forensic analysis. Spawner abundances were generally low and unpredictable in urban streams where the die-off phenomenon occurs. By contrast, in non-urban streams where coho are relatively abundant, spawners were unaffected. Therefore, tissue collections from coho in urban streams were ad hoc and opportunistic. The streams surveyed during the course of this study and associated samples collected are listed in Table 1.

**Table 1:** Summary of survey locations and associated tissue samples.

Location	Category	Years Sampled	Survey Frequency	Water Quality	Tissue Sampling				
					gills	brain	bile	pathogen screen	histopathology
**Longfellow Creek**	urban stream	2002–2009	daily	✓^b^	✓^c^	✓^a^	✓^a^^,^ b	✓^b^^,^ c	✓^b^^,^ c
**Piper's Creek**	urban stream	2006	daily						
**Des Moines Creek**	urban stream	2004	daily	✓^b^	✓			✓	✓
**Fortson Creek**	forested stream	2002	daily			✓	✓		
**University of Washington Hatchery**	urban hatchery	2002	one day			✓			
**Stillaguamish Hatchery**	rural hatchery	2002	one day			✓			
**Issaquah Creek Hatchery**	urban hatchery	2002, 2003	one day			✓^a^		✓	✓
**Wallace River Hatchery**	rural hatchery	2003, 2008	one day		✓^d^		✓	✓	✓
**Elliot Bay**	estuary	2003	one day				✓	✓	✓

a sampled only in 2002;

b sampled only in 2003;

c sampled only in 2004;

d sampled only in 2008.

To ensure sample integrity, tissues were not collected from the decomposing carcasses found in streams. Conversely, we did not sacrifice live, non-symptomatic fish in urban streams because these coho may or may not have survived to successfully spawn. As a consequence, some types of samples (e.g., gill metals, bile PAHs; see below) had to be collected from spawners that were either overtly symptomatic or very recently dead, as evidenced by gill coloration (see below). As noted earlier, symptomatic fish progress to death rapidly. Stream surveys were generally less than two hours in a given day, and thus encounters with symptomatic fish were infrequent. This accounts for a relatively small sample size for some tissues despite an intensive overall survey effort.

### Study locations

**Figure 1:**
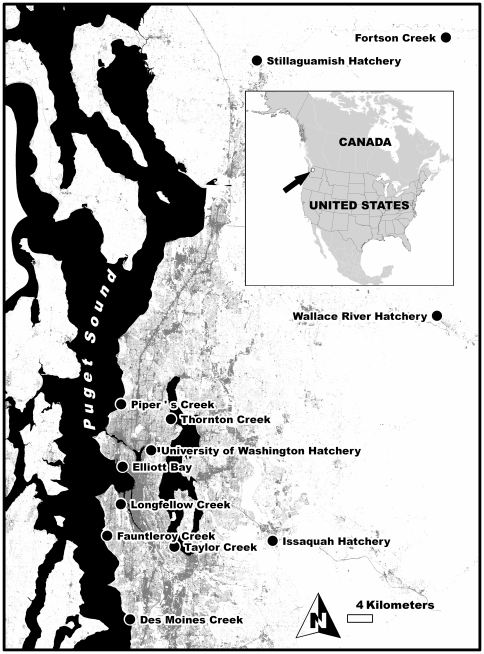
Stream survey and sample collection locations. The map indicates the greater Seattle metropolitan area, with gray shading representing the relative intensity of urbanization. Shown are the urban and non-urban creeks surveyed for coho spawner mortality, regional hatcheries, and the location of seawater-phase adult coho collections along the Seattle waterfront (Elliott Bay).

Daily or weekly spawner surveys (including tissue collections) were conducted on several Seattle-area streams from 2002–2009. These included Longfellow, Thornton, Piper's, Des Moines, Taylor, and Fauntleroy Creeks (Figure 1). Detailed descriptions of each of these drainages (except Des Moines) can be found in a recent City of Seattle report on urban waterways [7]. Longfellow Creek in West Seattle, the urban stream found to have the highest numbers of adult-entry coho in preliminary assessments (1999–2001), was the focus of daily surveys in each of the eight years of monitoring. Des Moines Creek to the southwest of Seattle was surveyed daily in 2004 and Piper's Creek in northwest Seattle was surveyed daily in 2006. In other years, these two urban streams were monitored approximately weekly, as were Taylor, Thornton, and Fauntleroy Creeks [8]. To assess the prevalence of pre-spawn mortality among wild coho salmon returning to spawn in a non-urban drainage, we surveyed Fortson Creek (a tributary to the North Fork Stillaguamish River north of Seattle; Figure 1) daily in the fall of 2002.

Tissue samples were also collected from regional hatcheries, including the Stillaguamish Tribal Hatchery (2002), the University of Washington Research and Teaching Hatchery (2002), the Wallace River State Hatchery (2003), and the Issaquah Creek State Hatchery (2002 & 2003). Salmon returning to both the University of Washington and Issaquah hatcheries traverse an urbanized landscape through a series of lakes separated from Puget Sound by a single set of locks. The Stillaguamish and Wallace River hatcheries are located on tributaries to the North Fork of the Stillaguamish River and the Snohomish River, respectively (Figure 1); returning coho pass through primarily rural and forested landscapes.

Adult seawater-phase coho salmon were collected in Elliott Bay (Seattle waterfront, lower Duwamish waterway) prior to freshwater entry. Animals were captured by gillnet in coordination with Muckleshoot tribal fishing operations in the early fall (September 11) of 2003. Adult coho were transferred live or recently dead (<3 hrs) to the NOAA research vessel *Harold W. Streeter* for immediate necropsy and sample storage.

### Spawner survey procedures

Coho typically return to Puget Sound urban streams in the early fall (i.e., early October), depending on the timing and intensity of rain events. Coho returned to the non-urban tributary of the North Fork Stillaguamish River (Fortson Creek) later in the fall relative to coho returning to urban creeks. Thus, daily surveys on Fortson Creek extended into late December in 2002 (approximately three weeks later than for surveys on urban creeks).

Daily surveys involved a visual inspection of most of the accessible freshwater habitat within a given stream. In some instances, poor visibility due to turbidity, high flows, or deep pools and wetlands precluded visual access. Surveyors began at the bottom of the reach and moved upstream, inspecting the stream channel for live adult salmon and carcasses. Where possible, the stream banks were also searched for carcasses that may have been dragged into the riparian zone by predators (predominantly river otters) or scavengers. Urban stream surveys spanned the entirety of the available spawning habitat within a given drainage. In some systems such as Longfellow Creek, impassable barriers restricted returning coho to spawning sites in the lower reaches of the stream.

**Figure 2:**
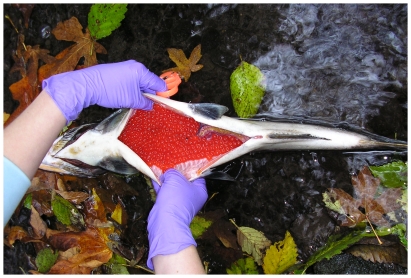
Representative adult female coho carcass with characteristically high egg retention. Rates of premature spawner mortality within and across urban drainages were quantified on the basis of egg-retaining female carcasses. Shown is a female affected by the mortality syndrome in Longfellow Creek in the fall of 2005. In most cases, egg retention was nearly 100%. Photo by Tiffany Linbo, NOAA Fisheries.

The location and general or atypical behavior of live salmon were recorded. For all dead or moribund fish, information on collection location, species, gender, fork length, weight (with and without ovaries for females), condition (e.g., signs of physical injury), and egg retention (females only) were recorded. Because males may spawn multiple times (or not at all) within a season, a determination of pre-spawn mortality was only made for females, and the reported rates of coho mortality for different streams are based on data from females only. Although we classified all female carcasses with >50% egg retention as pre-spawn mortalities, in most cases retention was closer to 100% (representative female in Figure 2). After examination, carcasses were left in the stream and marked with dated flagging tape. Even during high flow events, the day-to-day movement of flagged carcasses within streams was minimal (a few meters at most), and there was no evidence that pre- and post-spawn carcasses were disproportionately recoverable.

We collected tissue samples only from animals that were either overtly symptomatic or freshly dead (i.e., <3 hrs post-harvest or gill coloration was red to pink). Due to the limited availability of animals in some years, we collected tissues from symptomatic males as well as females.

Throughout the study we observed a mix of marked and unmarked fish returning to urban streams. In 2002, marked coho were identified by the absence of adipose fins. From 2003 through 2009, carcasses were scanned with a hand-held coded wire tag scanner (Northwest Marine Technology, Inc., Shaw Island, WA). To determine source hatcheries, retrieved tags were processed by U.S. Fish and Wildlife Service staff in the Fisheries Division of the Washington Fish and Wildlife Office (Lacey, WA).

### Fish condition

Condition factor was determined for pre- and post-spawn female coho salmon collected from Longfellow Creek (2002–09), Des Moines Creek (2004), and Fortson Creek (2002) as well as for female coho from the University of Washington and Stillaguamish Hatcheries (2002). Pre-spawn mortalities were weighed, the gonads removed, and then the fish were weighed again. All post-spawn and hatchery fish were weighed without gonads. Weight of the fish without gonads was used as a standard index to compare condition factor between pre- and post–spawn mortalities and hatchery fish. Condition factor was calculated using Fulton's condition factor (K = [weight (g)/length (cm)^3^]×100; [9]).

### Fish histopathology

Tissues were collected for histopathology in 2003 and 2004 from affected coho on Longfellow Creek (N = 21 animals) and Des Moines Creek (N = 22), healthy coho returning to the Wallace River (N = 20) and Issaquah (N = 24) hatcheries, and pre-freshwater entry coho from the Muckleshoot tribal fishery in Elliott Bay (N = 27). Samples of liver, head and trunk kidney, exocrine pancreas, pyloric caeca, small or upper intestine, large or lower intestine, stomach, heart, spleen, gonad, brain, and gill were preserved in Davidson's fixative [10]. Portions of each organ (3–7 mm in thickness) were excised from salmon bodies *in situ* and placed in 200 ml bottles filled with approximately 120 ml of fixative. Field-collected samples were transferred to the Northwest Fisheries Science Center, trimmed to a 3–4 mm thickness, and placed in tissue cassettes labeled with a unique fish identification number. Tissues were processed according to routine methods for paraffin embedding in Polyfin (Triangle Biomedical Sciences, Durham, NC) using a Shandon Hypercenter XP automated tissue processor (Shandon Lipshaw, Pittsburg, PA). Embedded sections were then cut to a 4–5 µm thickness, stained with hematoxylin and eosin-phloxine [11], and examined by light microscopy. Histopathologic diagnoses were coded as published previously [12].

The Fisher's Exact Test [13] was used to test whether the prevalences of certain histopathologic conditions in coho exhibiting pre-spawning mortality syndrome from the urban creeks were significantly higher than those for normal spawners from either the Wallace River or Issaquah hatcheries, or the pre-freshwater entry animals collected by gillnet from Elliott Bay. The critical level of significance was set at p≤0.05.

### Pathogen detection

Fish collected in 2003 and 2004 were screened for infectious non-viral pathogens commonly observed in Pacific salmon, especially those pathogens affecting osmoregulatory tissues such as trunk kidney and gill. In 2003, fish were analyzed for the myxosporean parasite *Parvicapsula minibicornis* (gill and kidney), the larval digenetic trematode parasite *Nanophyetes salmincola* (kidney), the bacterium *Renibacterium salmoninarum* (kidney), the myxosporean parasite *Tetracapsula bryosalmonae* (gill), the microsporidian parasite *Loma salmonae* (gill), and the myxosporean parasite *Ceratomyxa shasta* (posterior-most large intestine). In 2004, fish were analyzed for *P. minibicornis*, *R. salmoninarum*, *T. bryosalmonae*, and *L. salmonae*.

For *C. shasta* analysis, up to 5 mm of the posterior-most portion of the large intestine was placed immediately into extraction buffer [14] without proteinase K and placed on ice in the field. Within a few hours, samples were transported back to the lab and stored at 4°C. DNA extraction and analysis were completed by adding proteinase K and performing PCR with primers Cs1 and Cs3 by the method of Palenzuela et al. [14].

For *N. salmincola*, trunk kidney tissue was collected into a sample bag (Whirl-Pak™, Nasco, Modesto, CA) and immediately stored on ice. Within a few hours these tissues were transported back to the lab and stored at −20°C until analysis by light microscopy [15].

For *T. bryosalmonae*, *P. minibicornis*, *R. salmoninarum*, and *L. salmonae*, trunk kidney and gill tissues were placed into separate sample bags and immediately stored on ice. Within a few hours these tissues were transported back to the lab where they were stored at −20°C. DNA was extracted from approximately 25 mg of tissue using the Qiagen DNeasy tissue kit (Valencia, CA) with the following modification of the kit protocol. Samples were lysed in buffer (20 mM Tris-Cl, pH 8.0; 2 mM EDTA; 1.2% Triton X-100; 20 mg/ml lysozyme) for >45 m at 37°C and digested in 25 µl of proteinase K with 200 µl of buffer AL at 55°C overnight. Sample processing thereafter followed the kit protocol for animal tissues. PCR for *T. bryosalmonae* was performed with primers PKX5f and PKX6r [16] and for *P. minibicornis* with primers Parvi-1f and Parvi-2r [17]. *R. salmoninarum* was detected by nested PCR [18]. PCR for *L. salmonae* was performed with primers LS-1 and LS-2 [19]. To confirm the specificity of detection of these pathogens by PCR, representative amplification products were subjected to cycle sequencing by BigDye Terminator (version 3.1) reactions and analyzed on an ABI 3100 (Applied Biosystems, Foster, CA).

### Current use pesticide exposure (brain acetylcholinesterase activity)

Current use pesticides are commonly detected in urban streams [20]. Some of the most frequently detected insecticides, including diazinon, malathion, chlorpyrifos, and carbaryl are known to be neurotoxic to salmon [21], [22]. These chemicals inhibit the brain enzyme acetylcholinesterase (AChE), thereby disrupting chemical forms of synaptic communication in the salmon nervous system. Measures of brain AChE activity in fish are commonly used to diagnose anticholinesterase poisoning in response to insecticide exposure (e.g., [23]).

To assess the extent to which coho in urban streams may have been exposed to potentially toxic concentrations of insecticides, brains were collected from affected fish from Longfellow Creek (N = 32) as well as the non-urban reference stream (Fortson Creek; N = 18 pre-spawn, 38 post-spawn) and the Stillaguamish (N = 15 pre-spawn), University of Washington (N = 21 pre-spawn), and Issaquah (N = 21 pre-spawn) hatcheries. All samples were collected in the fall of 2002. Brains were dissected *in situ* from fresh carcasses (recently killed, or red to pink gills for fish collected from Longfellow Creek), flash frozen in liquid nitrogen, and then transported to the Northwest Fisheries Science Center for storage at −80°C. For analyses of AChE activity, tissues were homogenized in 50 mg ml^−1^ PBS-T (10 mM phosphate buffered saline containing 1% Triton X-100) and assayed on a 96-well plate using previously published methods for coho salmon [22]. Differences in brain AChE activity among locations were analyzed using a one-way ANOVA and Tukey-Kramer HSD posthoc test (JMP 8.0, SAS Institute, Inc., Cary, NC). The significance level was set at p≤0.05.

### Metal exposure

Gill tissue was collected opportunistically from affected coho from Longfellow Creek and Des Moines Creek in the fall of 2004 and from Wallace River Hatchery spawners in the fall of 2008. Samples were collected with plastic forceps and titanium scissors to avoid metal contamination. Upon collection, gill tissue was placed in plastic bags on ice in coolers and transported to the King County Environmental Laboratory (KCEL). Samples were stored at −20°C until analysis. Prior to analysis, samples were homogenized in blenders that were rinsed with methanol and wiped down prior to and between samples.

All samples were analyzed for arsenic, cadmium, chromium, copper, lead, nickel, and zinc. Total metals were measured by inductively coupled plasma-mass spectrometry (ICP-MS) using KCEL standard operating procedures. Tissue was digested with nitric acid in conjunction with hydrogen peroxide to remove the analytes from the sample matrix and then further digested in nitric and sulfuric acid in the presence of potassium permanganate and potassium persulfate. Sodium chloride hydroxylamine hydrochloride was added after digestion to reduce the sample and stannous chloride was added immediately before analysis.

Measures for quality assurance/quality control (QA/QC) included checking measurement accuracy against certified reference materials such as DORM-2 (dogfish muscle) from the Institute for National Measurement Standards (Ottawa, Canada). Further QA/QC procedures included the measurement of background metal levels with method blanks, monitoring variability with duplicate laboratory samples, and measuring recovery of total metals from spiked samples without (spike blank) and with (matrix spike) the sample matrix. Accepted variability for laboratory duplicates was 20%, ±15% for spike blanks, and ±25% for matrix spikes.

Gill tissue metal concentrations were normalized using a log_10_ transformation. For each metal, differences in concentrations due to location were analyzed using a one-way ANOVA and Tukey-Kramer HSD posthoc test with the level of significance set at p≤0.05.

### Polycyclic aromatic hydrocarbon exposure

Bile was collected from the gallbladders of returning adult coho during the 2002–2004 field seasons and analyzed for metabolites of polycyclic aromatic hydrocarbons (PAHs) using established methods [24]. Samples were collected from fish in an urban stream (Longfellow Creek in 2002 and 2003), a non-urban stream (Fortson Creek in 2002), a non-urban hatchery (Wallace River Hatchery in 2002), and seawater-phase fish prior to their entry into Seattle-area urban streams (Elliott Bay in 2003). Briefly, bile was injected directly onto a C_18_ reverse- phase column (Phenomenex Synergi Hydro, Torrance, CA) and eluted with a linear gradient from 100% water (containing a trace amount of acetic acid) to 100% methanol at a flow of 1.0 mL/min. Chromatograms were recorded at two fluorescence wavelength pairs: 1) 260/380 nm where several 3–4 ring compounds [e.g., phenanthrene (PHN)] fluoresce and 2) 380/430 nm where many 4–5 ring PACs [e.g., benzo[a]pyrene (BaP)] fluoresce. The concentrations of fluorescent aromatic compounds in bile were determined using PHN or BaP as external standards and converting the fluorescence response of bile to phenanthrene (ng PHN equivalents/g bile or ng PHN equivalents/mg protein) or benzo[a]pyrene (ng BaP equivalents/g bile or ng BaP equivalents/mg protein) equivalents. Total biliary protein was determined using the method of Fryer et al. [25]. Copper sulfate (in alkaline solution) and Folin reagent were added to each diluted bile sample (1∶1000 v:v with distilled water). The absorbance of each sample was measured at 620 nm using a plate-reader spectrophotometer and was compared to the absorbance of bovine serum albumin measured at this wavelength. Total biliary protein values are reported as mg protein/mL bile.

The HPLC/fluorescence system was calibrated prior to analyzing field samples by analyzing a PHN/BaP calibration standard numerous times (N = 5) until a relative standard deviation <15% was obtained for each PAC as previously described [26]. As part of the QA plan, a method blank and a fish bile control sample (bile from Atlantic salmon exposed to 25 µg of Monterey crude oil per mL of water for 48 hours) were analyzed with each salmon bile sample set.

Concentrations of PHN and BaP equivalents, as well as protein values, were log_10_-transformed to increase the homogeneity of variances. Analysis of variance (ANOVA) and the Tukey-Kramer HSD test were used to determine if mean concentrations of PHN and BaP equivalents and protein content of bile varied among collection years or collection sites. The level of significance was set at p≤0.05.

### Conventional water quality monitoring

Field meters were used to continuously monitor conventional water quality parameters during the fall of 2003 on both Longfellow Creek and Des Moines Creek. A 4a Minisonde™ (Hydrolab, Austin, TX) was installed by the City of Seattle in Longfellow Creek, in a pond at the terminus of the survey reach on this stream (just below an impassable culvert). A YSI 6600 multi-sonde unit (YSI Inc., Yellow Springs, OH) was installed by King County on Des Moines Creek. The unit was located in the stream channel below a footbridge in a community park, about 500 feet above the point at which the stream flows directly into Puget Sound. Both meters were programmed to measure and record water temperature, pH, dissolved oxygen, and specific conductance at 15-minute intervals. The Hydrolab was serviced and calibrated during the deployment period according to U.S. Geological Survey protocols [27]. The YSI meter was serviced weekly according to King County Environmental Lab standard operating procedures.

### Mortality in relation to rainfall patterns

We surveyed Longfellow Creek over eight consecutive years in part to evaluate the influence of rainfall on spawner mortality within and between fall coho runs. Daily and total rainfall data were collected as the sum of 1-minute interval detections from the nearest City of Seattle rain gauge (Rain Gauge 17, or RG17), located approximately 5 km southeast of the upstream terminus of the surveyed portion of the stream. Rainfall was quantified from one week prior to encountering the first live fish in Longfellow Creek until the day the last carcass was found. When data were not available at RG17 they were transposed from the next nearest rain gauge (RG18, distance approximately 13 km).

The relationship between inter-annual spawner mortality and total rainfall was assessed using binary logistic regression. For the correlation coefficient, the natural log of the odds ratio [(% pre-spawn/(1 - % pre-spawn)] for each year was weighted by sample size and regressed (simple linear regression) against total rainfall. In 2006, only 4 females were encountered, and data from this year (100% mortality) were excluded from the linear regression because they produced an undefined odds ratio. Both analyses were performed using JMP version 8 (SAS Institute Inc., Cary, NC).

## Results

### Behavior, condition, and origin of affected coho spawners

Consistent with initial observations of overtly symptomatic fish during early surveys in 1999–2001, we observed the same suite of behaviors in affected spawners during daily surveys from 2002–2009. These included circular surface swimming (loss of orientation), gaping, pectoral fin splaying, and loss of equilibrium (Video S2, S3). Symptomatic coho encountered during the course of a survey usually died by the end of the survey (i.e., within 1–2 hrs). Those that were still alive were found as pre-spawn carcasses the next day. Symptoms were displayed by both male and female spawners, were consistent from year to year, and were consistent across urban drainages.

Numerous adult coho carcasses were found in all monitored streams (Figure 1 and Table 2). For the urban streams, the frequency of egg retention among dead females (Figure 2) was high. For example, for Longfellow Creek, pre-spawn mortality ranged between 70–90% of the overall run in years where returning coho were relatively abundant (Table 2).

**Table 2:** Rates of coho pre-spawn mortality in Puget Sound lowland streams surveyed daily from 2002–2009.

Creek	Year	N*	% Wild**	% Pre-Spawn Mortality Wild	% Pre-Spawn Mortality Total
**Longfellow**	2002	57	4	100	86
	2003	18	28	20	67
	2004	9	89	88	89
	2005	75	72	72	72
	2006	4	75	100	100
	2007	41	10	75	73
	2008	12	0	n.a	67
	2009	44	0	n.a.	79
**Piper's**	2006	9	78	100	100
**Des Moines**	2004	30	33	60	63
**Fortson (non-urban)**	2002	114	100	0.9	0.9

*Sample size reflects female coho of known spawning condition, with no signs of predation.

**Presumed wild origin based on presence of adipose fin and absence of a coded wire tag.

The size and condition of affected fish from urban streams were comparable to those of wild coho returning to Fortson Creek and unaffected hatchery coho returning to regional hatcheries (Table 3). For example, the condition factor for affected fish from Longfellow Creek in 2002 was not significantly different than the condition factor for wild coho from the non-urban reference stream (Fortson Creek; t-test, p = 0.12) and it was higher than the condition factors for the unaffected hatchery fish (e.g., Issaquah Hatchery; Table 3).

**Table 3:** Spawner condition for female coho salmon collected from urban and non-urban locations.

Site	Year	N	Mean Condition*	SD
**Fortson Creek**	2002	20	0.893	0.111
**University of Washington Hatchery**	2002	21	0.816	0.103
**Stillaguamish Hatchery**	2002	5	0.840	0.047
**Issaquah Hatchery**	2002	21	0.814	0.06
**Longfellow Creek**	2002	47	0.856	0.077
	2003	10	0.920	0.137
	2004	8	1.018	0.103
	2005	54	1.057	0.105
	2006	4	1.084	0.078
	2007	21	0.995	0.153
	2008	7	1.032	0.122
	2009	30	0.930	0.235
**Des Moines Creek**	2004	19	1.109	0.268
**Piper's Creek**	2006	9	1.055	0.107

*Condition Factor was Fulton's K = (weight/(length∧3))*100. Weights were for gravid females (i.e., with ovaries containing eggs).

The spawner mortality syndrome appears to be specific to coho in urban drainages. We observed no symptoms and less than 1% pre-spawn mortality among wild coho returning to spawn in the non-urban reference stream in 2002 (Fortson Creek; Table 2). This is consistent with a widely reported absence of the syndrome among coho spawners from non-urban catchments. For example, in 2003–2004, Washington Trout (now the Wild Fish Conservancy) surveyed 29 Washington Department of Fish and Wildlife index reaches (<10% developed land cover) for coho spawner success in the Snohomish River basin north of Seattle. Of more than 1,000 intact adult female carcasses inspected, less than 0.5% died with an egg retention rate of >50% [28].

We did not observe corresponding die-offs of resident fish in urban streams (e.g., sticklebacks, sculpins, or cutthroat trout), nor did we find the syndrome in other species of migratory salmon return to these same urban streams to spawn in the fall. Also, the phenomenon appears to be specific to adult coho. In 2003, water from Longfellow Creek was diverted into a flow-through streamside shed facility with juvenile coho housed individually in separate aquaria (N = 24). The juveniles were fed daily and monitored throughout the duration of the fall spawner run. Despite the presence of symptomatic adults in the adjacent stream, juveniles exposed to the same surface flows showed no overt symptoms, with 100% survival across the experimental group (data not shown).

Throughout the study, the general dearth of coho salmon returning to Seattle-area urban streams posed a challenge in terms of collecting tissues for forensic analyses. Longfellow Creek was chosen as a site for long-term monitoring in part because of the proportionally higher number of coho that typically enter this drainage relative to the other urban creeks in Seattle. Coded wire tag analysis of >50 tags collected from coho in Longfellow Creek (2003–2008) showed that many of these fish are hatchery strays originating from a net pen facility operated in Elliott Bay by the Muckleshoot and Suquamish Tribes. This facility serves to transition approximately 500,000 juvenile coho each year from the Soos Creek hatchery (Washington State Department of Wildlife) to the saltwater environment. Importantly, however, for certain high-return years (e.g., 2002 and 2005: Table 2), most of the stricken coho spawners were unmarked and presumably of wild origin. The mortality syndrome therefore appears to affect wild and hatchery coho alike.

### Coho mortality is not correlated with common pathogen-associated disease or noninfectious lesions

A systematic survey of histopathological conditions in pre-spawn carcasses (urban streams) and unaffected fish from Elliott Bay and two regional hatcheries (Wallace River and Issaquah Creek) was conducted in 2003 and 2004. Various infectious, parasitic and idiopathic (of unknown etiology) diseases were detected in the gill, heart, trunk kidney, gastrointestinal tract and liver of adult spawners. These findings are summarized in Table S1 and Text S1. No significant lesions were detected in the gonad, brain, spleen or exocrine pancreas of adult coho regardless of origin. None of the observed disease conditions were specific to animals that succumbed to the mortality syndrome, nor were they unique to the urban streams where die-off rates were high.

Among the six pathogens screened by PCR or microscopy, *L. salmonae* in the gill and *R. salmoninarum* in the kidney were detected among fish from all sampling sites, with the highest prevalence observed among Longfellow Creek fish (*L. salmonae*) or Des Moines Creek fish (*R. salmoninarum*) (Table S1). In contrast, *P. minibicornis* in the gill or kidney was detected among fish from all sites except Des Moines Creek. Prevalence of *T. bryosalmonae* in kidney, *N. salmincola* in kidney, and *C. shasta* in lower intestine varied widely among the sites, ranging from 0% to 48%. Although no single pathogen was consistently associated with pre-spawn mortality, infection or infestation by multiple pathogens occurred more frequently among Longfellow Creek fish. In 2003 fish from Longfellow Creek exhibited a significantly higher number of infections or infestations per fish (median = 3) than fish from Elliott Bay (median = 2), Issaquah Hatchery (median = 1), or Wallace River Hatchery (median = 1) (Fisher Exact Probability, p<0.0001; Figure S1).

### Prematurely dying spawners show no indication of exposure to common insecticides

**Figure 3:**
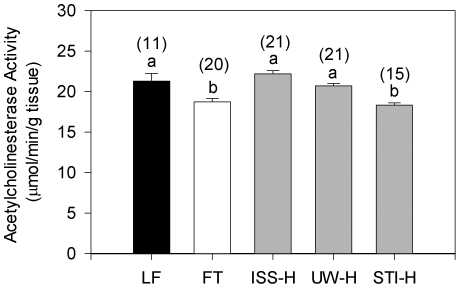
Prematurely dying spawners do not show evidence of neurotoxic pesticide exposure. Shown are relative rates of brain acetylcholinesterase (AChE) activity, a target enzyme for common homeowner use insecticides, in adult coho salmon. The brain enzyme activities of affected fish from an urban stream (Longfellow Creek; LF) were not significantly inhibited relative to unaffected fish from a non-urban stream (Fortson Creek; FT) and three regional hatcheries; Issaquah (ISS-H), University of Washington (UW-H), and Stillaguamish (STI-H). Error bars are 1 standard error of the mean. Sample size is indicated in parentheses and letters indicate significant differences between locations (one-way ANOVA, Tukey's HSD; p<0.05).

A reduction in the enzymatic rate of brain acetylcholinesterase is a bioindicator of exposure to common carbamate and organophosphate insecticides. As shown in Figure 3, measured brain enzyme activity affected coho spawners from Longfellow Creek was not significantly lower than corresponding brain enzyme activities in fish from a non-urban reference stream (Fortson Creek) or from regional hatcheries. Rather, AChE activity in the brains of affected fish was slightly but significantly higher than for coho spawners from Fortson Creek and the Stillaguamish Tribal Hatchery (one-way ANOVA; p<0.05).

### Affected coho have elevated levels of metals in gill tissues

**Figure 4:**
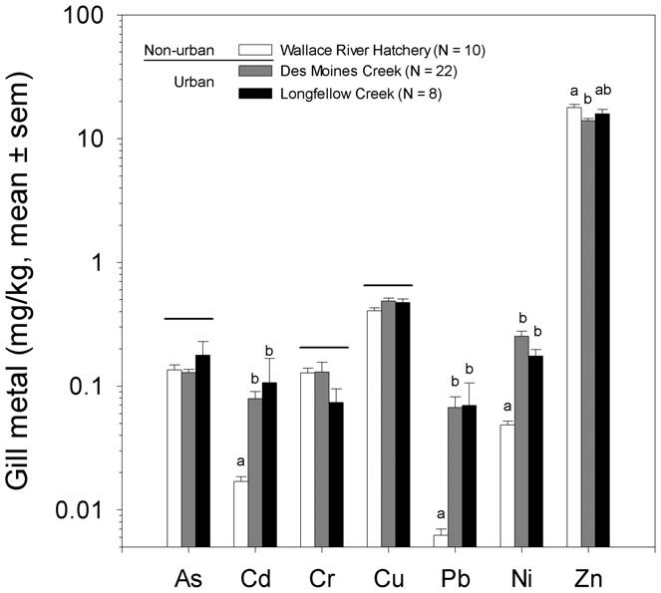
The gill tissues of prematurely dying coho contain elevated levels of cadmium, lead, and nickel. The concentrations of metals in the gills of affected coho from two urban streams (Longfellow and Des Moines Creeks) are plotted relative to samples collected from a non-urban hatchery (Wallace River). Error bars are 1 standard error of the mean. Letters indicate significant differences in measured levels of metals between sampling locations (one-way ANOVA, Tukey's HSD; p<0.05) and horizontal bars indicate no significant differences (p>0.05).

The measured concentrations of arsenic, cadmium, chromium, copper, lead, nickel, and zinc in the gill tissue of adult salmon collected from two urban sites (Des Moines and Longfellow Creeks) and one non-urban site (Wallace River Hatchery) are shown in Figure 4. There were significant differences among the three sites for cadmium, lead, nickel, and zinc (one-way ANOVA and Tukey-Kramer HSD; p<0.05). Fish from the two urban streams had similar levels of cadmium, lead, and nickel, and these were significantly higher than corresponding levels in the gills of coho from the non-urban location. For zinc, fish from the non-urban hatchery had slightly but significantly higher gill concentrations relative to fish from one of the two urban streams (Des Moines Creek).

### Bile analysis indicates elevated exposure to petroleum hydrocarbons in fish from urban streams

**Figure 5:**
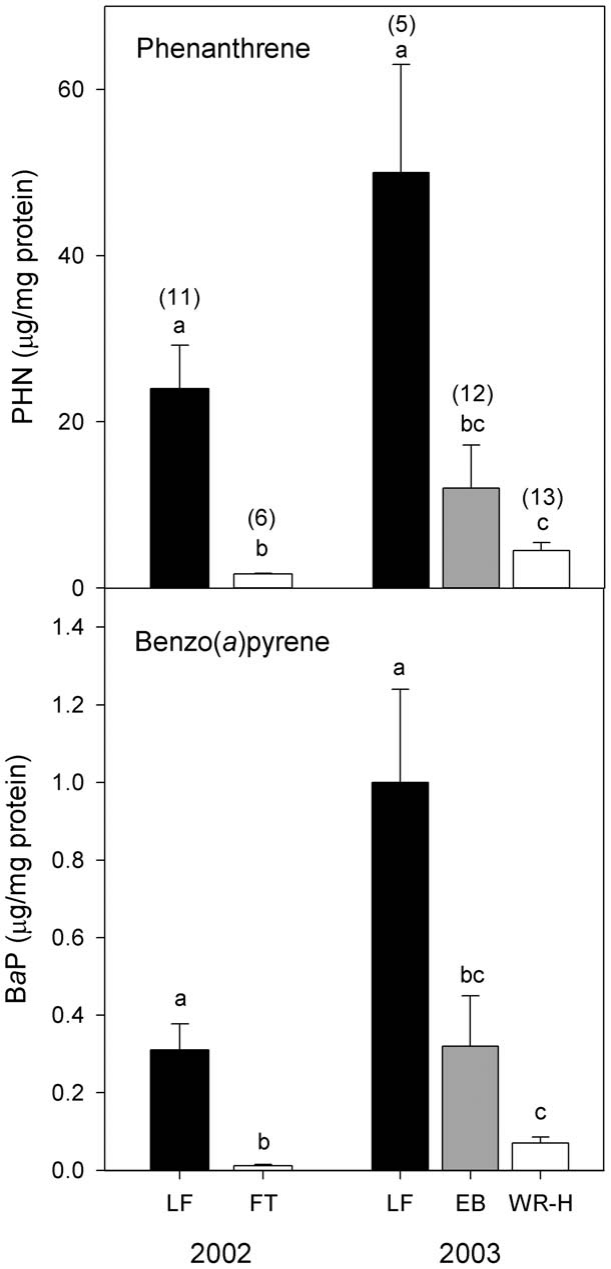
Analysis of bile from affected coho spawners indicates exposure to polycyclic aromatic hydrocarbons (PAHs). Concentrations of fluorescent PAH metabolites (as phenanthrene [PHN] and benzo-*a*-pyrene [BaP] equivalents) in the bile of coho collected in an urban stream (Longfellow Creek; LF) are shown relative to fish from a non-urban stream (Fortson Creek; FT) and a non-urban hatchery (Wallace River Hatchery; WR-H). In 2003, seawater-phase coho (pre-freshwater entry) were also sampled from urban Elliott Bay (EB). The bile data demonstrate a significant increase in PAH exposure after coho spawners transition from a highly urbanized estuary to freshwater spawning habitats. Sample sizes, the same for PHN and BaP, are indicated over each bar. Error bars are 1 standard error of the mean.

In the fall of 2002, bile was collected from symptomatic and recently dead coho from Longfellow Creek, as well as from adults returning to spawn in the non-urban reference stream (Fortson Creek). Relative exposures to polycyclic aromatic hydrocarbons (PAHs) were quantified by measuring mean concentrations of phenanthrene (PHN) and benzo(a)pyrene (BaP) metabolites in bile. As shown in Figure 5, affected fish from the urban stream had significantly higher biliary levels of both PHN and BaP equivalents (one way ANOVA, Tukey-Kramer HSD, p<0.05).

In 2003, the biliary levels of PAHs in fish from Longfellow Creek were compared to PAH levels in seawater-phase adults collected from a gillnet fishery in Elliott Bay, prior to freshwater entry, and adults returning to a non-urban hatchery (Wallace River Hatchery). As in 2002, fish from the urban stream showed significantly higher exposures to both PHN and BaP relative to the non-urban sampling location (Figure 5; one way ANOVA, Tukey-Kramer HSD, p<0.05). PAH levels in the bile of seawater-phase coho collected from Elliott Bay were slightly but not significantly elevated relative to the non-urban location.

### Stream temperature, dissolved oxygen, and other conventional water quality parameters do not appear to be causal factors for the mortality syndrome

Monitoring results for conventional surface water quality parameters in urban drainages where premature coho mortality is prevalent have been published previously [7]. During the fall months, urban streams were cool and well mixed. For example, fixed station surface temperature monitoring on Longfellow Creek between October and December in 2002 (86% pre-spawn mortality across the entire run that year; Table 2) revealed maximum daily temperatures ranging from about 6–11°C. Over the same interval, surface water concentrations of dissolved oxygen ranged from about 9–11 mg/L. This fall and winter pattern of relatively cool, oxygen-rich surface flows is also typical of other urban streams where coho die-offs commonly occur (e.g., Piper's Creek; [7]). Monitored conditions for other conventional water quality parameters were also favorable for salmon health and survival. The average level of ammonia-N in water samples collected from Longfellow Creek during storm events (0.04 mg/L) was more than an order of magnitude below the pH-adjusted benchmark criterion for chronic ammonia toxicity (0.43–2.1 mg/L). Moreover, pH levels were normal (pH 6.5–8.5) for Longfellow Creek in the survey years included in this study [7].

### Mortality is qualitatively but not quantitatively influenced by rainfall

**Figure 6:**
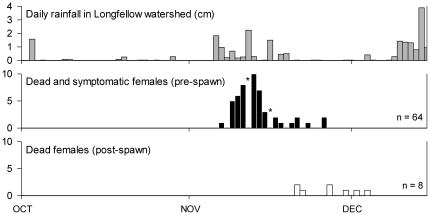
Pre-spawn mortality and survival to spawn in relation to rainfall. Shown are the results of daily stream surveys throughout the 2002 coho spawning season in Longfellow Creek in relation to daily rainfall. Asterisks (*) indicate days when stream flows were too high to survey the creek.

During the first year of annual surveys on Longfellow Creek (2002), fall coho returns were several weeks late due to an unusually dry October and early November. The first significant rains in the second week of November triggered a large influx of spawners. As the rains continued over the next two weeks, every fish entering the drainage succumbed to the mortality syndrome, with many observations of overt symptomology during daily surveys (Figure 6). Fish only survived to spawn in the weeks following the mid-November storms.

Based in part on the apparent strength of this association between rainfall and mortality in 2002, we continued with daily surveys on Longfellow Creek in successive years (2003–2009). The relationship between inter-annual variation in total rainfall and the severity of spawner mortality was evaluated using binary logistic regression. The results suggest a pattern of higher coho survival in wetter years where more water moves through the watershed before many of the adults arrive on the spawning grounds. However, there was a large amount of inter-annual variability in both rainfall (timing and amount) and coho returns (timing and number). As a consequence, the logistic regression was not significant at p≤0.05 (not shown; χ2(1) = 1.70, p = 0.19). The slope for the regression was −0.021 (se = 0.016, p = 0.19) and the intercept was 1.674 (se = 0.403, p<0.0001). Across years, rainfall explained 29% of the variability in the spawner mortality syndrome (log odds ratio weighted linear regression, r^2^ = 0.288).

Notably, in each of the eight survey years, the first carcass found was always a pre-spawn mortality. Conversely, in six of the seven years in which at least one fish survived to spawn, the last carcass found was a successful spawner.

## Discussion

We have documented a distinct mortality phenomenon among adult coho salmon returning to spawn in urban watersheds of central Puget Sound. The syndrome has been recurrent for more than a decade, with a consistent symptomology across years and survey locations. The annual die-offs have claimed a large proportion of the fall runs in the drainages monitored during the course of this study. These high mortality rates (e.g., 60–100%) are likely to preclude sustainable natural production in urban drainages more generally [29], and the coho we monitored during the course of this study were fish that appear to have strayed into ecological traps [30] in search of spawning habitat. Our findings fit a general pattern for Puget Sound, in which adult coho are very few in number in watersheds where the mortality syndrome has been observed. Conversely, in (non-urban) watersheds where coho spawners are relatively abundant, they appear to be unaffected.

Factors that are known to cause spawner mortalities in other species of salmon do not appear to be involved in the coho pre-spawn syndrome that we have explored here. The temperatures and dissolved oxygen content of urban streams during mortality events were not unusually high or low, respectively. Although all fish of the fish we examined showed evidence of infection with common pathogens, there was no correlation with the high rates of mortality in urban drainages or the observed symptomology. Lastly, the stricken coho were generally in good physical condition, and we found no evidence that origin (i.e., wild or hatchery) influences an animal's susceptibility.

The weight of evidence therefore suggests that adult coho salmon are unusually vulnerable to the toxic effects of one or more chemical contaminants, most likely delivered to urban spawning habitats via stormwater runoff. The rapid progression of the syndrome and the specific nature of the symptoms are consistent with acute cardiorespiratory toxicity. Our current findings support this hypothesis, albeit indirectly by ruling out alternative, non-chemical explanations.

We found that affected coho show elevated exposure to metals and petroleum hydrocarbons, the latter after spawners transition to freshwater from a highly urbanized estuary (Elliott Bay). Evidence of exposure to metals and PAHs does not imply causality, but future studies should address these toxics, as they are specifically known to disrupt respiratory, osmoregulatory, and cardiovascular physiology in fish. The abrasion of vehicle tires and brake pads releases aluminum, barium, cadmium, cobalt, copper, lead, nickel, zinc, and other elements onto impervious surfaces [31]. Copper and other metals disrupt ionoregulation via binding to ligands in the fish gill [32] without causing overt cytotoxicity. Moreover, metals are generally more bioavailable and thus more toxic to fish in soft waters such as stormwater [33]. Motor vehicles are also sources of PAHs via exhaust and leaking crankcase oil. Certain PAHs are cardiotoxic to fish [34], including specifically the tricyclics phenanthrene and dibenzothiophene (e.g., [35]).

It is important to note, however, that the toxicological context (i.e., established literature) for anticipating possible acutely lethal toxic effects of stormwater contaminants on coho spawners is practically nonexistent. On the one hand, urban runoff typically contains organic chemicals and metals in the low parts per billion to parts per trillion range (e.g., [36], [37]), well below levels that would be expected to cause fish kills based on established median lethal (LC_50_) concentrations for rainbow trout and other salmonids. On the other hand, to our knowledge, there have been no toxicological studies on freshwater-transitional adult coho. When adults return from saltwater to freshwater in preparation for spawning, they undergo osmoregulatory adjustments that include shifts in plasma osmolality, gill sodium-potassium ATPase activity, and the density of chloride cells in the gill (e.g., [38]), as well as changes in the circulation of stress and reproductive hormones [2]. These changes may render adult animals particularly vulnerable to toxics that interfere with the physiological processes that underlie freshwater acclimation. Coho have recently been shown to be considerably more vulnerable to chemical toxicity when they make the opposite transition from freshwater to saltwater [39].

Sensitivity related to freshwater transition might explain our observations of affected adults and unaffected juveniles exposed to the same surface waters, but not our observations of affected coho spawners side-by-side with unaffected spawners of other salmon species. For example, in 2006 there were temporally overlapping runs of coho and chum spawners in Piper's Creek. Whereas all of the coho succumbed, the egg retention rate for chum carcasses was <4% (5 of 135 females; data not shown). Moreover, symptomatic coho were observed in the stream side-by-side with healthy chum, the latter actively digging and defending redds (Video S4). The underlying reason for the interspecific difference in sensitivity remains to be determined.

More work is also needed to define the influence of rainfall on the spawner mortality syndrome. The clearest indication that rainfall plays a role was the 2002 survey results for Longfellow Creek. That year was characterized by an unusually long antecedent dry interval (presumably allowing a proportionally greater accumulation of pollutants on impervious surfaces within the watershed), a relative abundance of returning spawners, and consistent rainfall for approximately two weeks at the beginning of the compressed run. As in 2002, in subsequent years we observed a general tendency towards higher survival later in the run, after multiple fall rain events. However, the relationship was not statistically significant across the survey years, due in part to highly variable rainfall patterns, longer run durations, and very low spawner numbers in some years. Notably, the mortality syndrome is not a simple first-flush phenomenon, as spawned and unspawned carcasses were usually intermixed throughout the duration of each run in 2003–2009.

Additional evidence implicating urban runoff was recently provided by a spatial land use analysis of the watersheds surveyed during the course of this study. Feist et al. [40] found that inter-watershed rates of coho spawner mortality correlate closely and positively with the relative proportion of local roads, impervious surfaces, and commercial property within a basin. These and other correlations were then used to predict areas of possible coho spawner die-offs in unmonitored drainages throughout central Puget Sound [40]. The link to roads and other impervious surfaces further implicates motor vehicles as the likely source of causal toxics, but this remains to be demonstrated directly – e.g., by reproducing the mortality syndrome in otherwise healthy adult coho via exposures to environmentally relevant mixtures of metals and PAHs in freshwater.

Recent population-scale modeling has shown the potential for rapid local declines in coho population abundance across the range of spawner mortality rates observed in urban drainages during the course of this study [29]. Regional human population growth and land use changes that increase the proportion of impervious surfaces within watersheds may therefore pose an important future threat to wild coho populations if 1) toxic urban runoff is the underlying cause of the mortality phenomenon, and 2) wild coho are similar in their vulnerability to the hatchery and unmarked (and presumably wild) coho that were found unspawned in 2002–2009. This is above and beyond the established and widely documented stormwater-driven threats to aquatic habitats (e.g., [41], [42], [43]).

In closing, past efforts to restore salmon habitats in Seattle-area urban watersheds have revealed unexpected challenges for improving coho spawner abundance and survival. These restoration projects have been successful in numerous other ways, including revitalizing urban green spaces, extending watershed connectivity, enhancing public education and involvement, and improving habitat conditions for otters, waterfowl, amphibians, stream invertebrates, native plants, and other fish species. Restored urban streams have also provided an experimental setting to study what may become a very important threat to wild coho populations in the decades ahead as some healthy stream networks gradually acquire the land cover characteristics of the Longfellow Creek system and similar urban drainages. The next generation of urban watershed improvements is now underway, including the catchment-scale implementation of natural drainage systems (using green infrastructure and other emerging technologies), floodplain restoration, and new pollution mitigation activities such as vacuum sweeping of roadways. Moreover, Washington recently became the first state in the U.S. to legislatively mandate a phased reduction of metals in vehicle brake pads and other friction materials (SB6557). Future improvements in the survival of adult coho in urban streams will be an important indicator of success for these and other pollution reduction strategies.

## Supporting Information

Text S1 A detailed description of the histopathology results from tissue samples collected during the study from urban and non-urban sites.
(DOC)

Figure S1 Number of pathogens per fish detected by pathogen screening methods for fish collected in 2003. Horizontal bar is positioned at the median. Longfellow Creek fish differ from fish from all other locations (Chi-square test, *p*<0.0001).
(TIFF)

Table S1 Prevalence of infectious (parasitic/bacterial) and idiopathic conditions detected by histopathology and by pathogen screening (molecular and microscopic) in adult coho salmon sampled from several creeks and hatcheries in the Puget Sound region in 2003 and 2004. H = histopathology; PS = pathogen screening methods; − = analysis not performed.
(DOC)

Video S1 Symptomatic female coho salmon in Piper's Creek in 2000. The fish appears to be in good physical condition, with ocean-bright (silver) coloration. Characteristic symptoms include loss of equilibrium, gaping, and pectoral fin splaying.
(MP4)

Video S2 Early onset symptomology of an affected adult coho salmon in Longfellow Creek in 2002. The fish has lost orientation (surface swimming) and is gaping.
(MP4)

Video S3 Late-stage symptomology of an affected adult coho salmon in Longfellow Creek in 2002. The fish has lost equilibrium and is gaping, with pectoral fins splayed.
(MP4)

Video S4 A symptomatic coho spawner in Piper's Creek in 2006, just downstream of unaffected chum spawners displaying normal spawning behavior.
(MP4)
