# Supplementary material for: Recurrent Die-Offs of Adult Coho Salmon Returning to Spawn in Puget Sound Lowland Urban Streams
Source: PLoS One. 2011 Dec 14;6(12):e28013. doi: 10.1371/journal.pone.0028013 (PMC3237429; doi:10.1371/journal.pone.0028013)
Supplement: Text S1 — A detailed description of the histopathology results from tissue samples collected during the study from urban and non-urban sites. (DOC) [file pone.0028013.s001.doc]

**Supplemental Data**

In the gill, coho from all sites except Elliott Bay were lightly infected with the external ciliate *Ichthyophthirius* *sp*., exhibiting the life history stages of tomites or trophonts. Prevalences ranged from 18% at Des Moines Creek to 58% at the Wallace River hatchery (refer to Table S1 for histopathology and pathogen data). Fish from all sampling sites were infected with the intracellular microsporidan parasite, *Loma salmonae*, typically exhibited as small, multifocal xenomas within the connective tissue of the gill filaments and lamellae. Infections by *L. salmonae* were typically very light, and with a low intensity host response to these protozoan parasites. Prevalences of *L. salmonae* by histopathology ranged from 5% at the Wallace River hatchery to a high of 24% at Longfellow Creek. Prevalence by pathogen screening with PCR specific for *L. salmonae* approximated the histopathology prevalence at each site. Coho from only Elliott Bay (41%) showed infestations by a monogenetic trematode. The idiopathic conditions represented by respiratory epithelial hyperplasia and microaneurysms were also detected at variable prevalences at most of the sites, but not at prevalences that were higher at the urban creeks where pre-spawn mortality was present. The prevalence of *Parvicapsula* *minibicornis* ranged from 0% (Des Moines Creek) to 100% (Wallace River Hatchery), with no obvious association with urban sites.

In the heart, *L. salmonae* was also detected at highest prevalence in coho from Longfellow Creek (19%), with slightly lower prevalences at Des Moines Creek, Issaquah hatchery, and Wallace River hatchery. The larval trematode *Nanophyetus salmincola* (commonly referred to as the salmon poisoning fluke) was also detected at low prevalences (0-5%) at the various sampling sites.

In the trunk kidney, myxosporeans consistent with the morphology of *Myxidium spp*. infected the tubular epithelium and lumina of coho nephrons from all sites except Elliott Bay, with the highest prevalences at Wallace River hatchery (85%) and Longfellow Creek (67%). The stages most commonly seen were the trophozoite stages, making definitive diagnosis in all cases problematic since spores could not be found and identified in all of the cases. There was very little pathology or host response associated with these myxosporeans. The prevalence of *P. minibicornis* detected by species-specific PCR was greater than 65% except among Des Moines Creek fish (0%). Other renal infections detected among the sampling sites included low prevalences of *N. salmincola*, *L. salmonae*, *Ceratomyxa spp*., *Tetracapsula* *bryosalmonae*, and *Renibacterium salmoninarum*. The prevalence of *N. salmincola* was similar at each site between the histopathology and pathogen screening methods, with no infestation detected at either hatchery site by both methods, and slightly higher prevalence at the Longfellow Creek and Elliott Bay sites by the pathogen screening method. *T.* *bryosalmonae* prevalence was not detected or was less than 10% at all sites except at the Wallace River Hatchery site (40%). Infection with *R. salmoninarum* was observed at all sites, ranging from 22% (Elliott Bay) to 100% (Des Moines Creek). None of these infections or infestations were associated with fish exhibiting the pre-spawn mortality syndrome.

An idiopathic condition detected primarily at Des Moines Creek (52%) was characterized by protein casts within the tubular lumina, consistent with nephrosis. However, this lesion can also closely mimic autolytic postmortem artifactual changes in the fish kidney, which may explain the higher prevalence in fish from this site in 2004, since such a high proportion of these fish were sampled postmortem.

Several parasitic conditions were also detected in the organs of the gastrointestinal tract (stomach, pyloric caeca, upper and lower intestine) at varying prevalences in coho from all of the sampling sites. These included a mucosal infection by *Ceratomyxa spp*. (probably *Ceratomyxa shasta*), which was especially prevalent at the Issaquah hatchery; cestodes and digenetic trematodes in the gastrointestinal lumen; nematodes in the mesenteric tissue associated with the gastrointestinal tract; and a single case of an unidentified myxosporean in the gastrointestinal mucosa of a coho from Des Moines Creek. Prevalence of *C. shasta* in the posterior-most portion of the gastrointestinal tract detected by species-specific PCR was less than 5% except among fish from Elliott Bay (18%). None of these parasites showed higher prevalences in coho exhibiting the pre-spawn mortality syndrome.

Several idiopathic lesions were detected in the liver of sampled coho from most of the sites, generally at higher prevalences in pre-spawn coho from Longfellow and Des Moines Creeks. Specifically, hydropic degeneration and/or hepatocellular coagulative necrosis were detected in 25% and 36% of pre-spawn coho from Longfellow Creek and Des Moines Creek, respectively, as compared to 0% at Elliott Bay, 19% at Issaquah hatchery, and 17% at Wallace River hatchery. Similarly, the unique hepatic conditions of hepatocellular megalocytosis and nuclear pleomorphism were detected at slightly higher prevalences in coho from Longfellow (15%) and Des Moines (9%) Creeks, as compared to 6% at Wallace River hatchery and 0% at Elliott Bay and Issaquah hatchery. These observations are particularly noteworthy since the latter lesions of hepatocellular megalocytosis and nuclear pleomorphism have been consistently associated with exposure to PAHs in wild English sole and other species (Myers and Fournie 2002, Myers et al. 2003) and have been induced in the laboratory by exposure of English sole to PAHs (Schiewe et al. 1991).

The distribution of prevalences of infectious, parasitic and idiopathic conditions detected among the sites suggested that some of these conditions might be associated with the pre-spawn mortality syndrome (Table S1), although in no case was there a condition that was always present in pre-spawn coho from the urban creeks and always absent in normally spawning coho from the hatcheries and Elliott Bay. For example, the prevalence of *L. salmonae* in any organ was highest (29%) in Longfellow Creek, as compared to lower prevalences at sites not exhibiting pre-spawn mortality (7-17%). In fact, the prevalence of this microsporidan at Longfellow Creek for 2003-4 approached being significantly higher (p = 0.06) by the Fisher’s Exact Test as compared to the affected/unaffected ratio in combined coho from Elliott Bay and the hatcheries. However, if the prevalence data for pre-spawn coho from Longfellow and Des Moines Creek are combined and compared to the other sites with normally spawning coho, there are no significant differences in prevalence. Furthermore, the fact that none of the eight pre-spawn coho sampled in 2004 from Longfellow Creek were infected with *L. salmonae*, combined with the typically low intensity infection manifested in the gills and hearts of pre-spawn coho, argues strongly against this infectious agent being a major factor in contributing to the pre-spawn mortality syndrome.

The relationship between the occurrence of multiple pathogens and collection site was examined. Fish collected in 2003 (but not in 2004) were screened for one gastrointestinal pathogen, three kidney pathogens, one gill pathogen, and one pathogen in both kidney and gill, for a maximum of six pathogens per fish. The median number of pathogens per fish was higher at Longfellow Creek (three per fish) than at the remaining sites (Figure S1), and was significantly higher among Longfellow Creek fish when the number of pathogens per fish were binned by one or two per fish and three or four per fish (Chi-square, p < 0.0001),

The idiopathic hepatic conditions detected at higher prevalences in pre-spawn coho show statistically higher prevalences at the urban creeks than in coho from the other sites combined (Table S1). For example, coho were affected by hydropic degeneration and hepatocellular coagulative necrosis at a higher prevalence at Des Moines Creek (p = 0.0095), and at the combined sites of Longfellow and Des Moines Creeks (p=0.0174) than at the other sites where pre-spawn coho mortality did not occur. Likewise, hepatocellular megalocytosis and nuclear pleomorphism was also significantly higher at Longfellow Creek (p = 0.0377) and at the combined sites of Longfellow and Des Moines Creeks (p = 0.0321). However, because the prevalences of these idiopathic conditions were overall rather low in the pre-spawn coho and the lesion severity was typically mild to moderate, it is not likely that these conditions contributed significantly to the death of the affected fish; the toxicologic relevance of these lesions as histopathologic biomarkers of PAH exposure and effect is probably more meaningful.
