# Supplementary figures and images for: Recurrent Die-Offs of Adult Coho Salmon Returning to Spawn in Puget Sound Lowland Urban Streams
Source: PLoS One. 2011 Dec 14;6(12):e28013. doi: 10.1371/journal.pone.0028013 (PMC3237429; doi:10.1371/journal.pone.0028013)

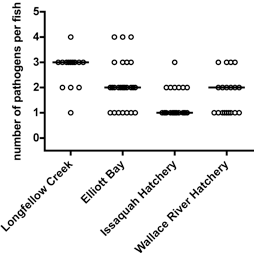

Supplement: Figure S1 — Number of pathogens per fish detected by pathogen screening methods for fish collected in 2003. Horizontal bar is positioned at the median. Longfellow Creek fish differ from fish from all other locations (Chi-square test, p<0.0001). (TIFF) [file pone.0028013.s002.tif]
