# Supplementary material for: Recurrent Die-Offs of Adult Coho Salmon Returning to Spawn in Puget Sound Lowland Urban Streams
Source: PLoS One. 2011 Dec 14;6(12):e28013. doi: 10.1371/journal.pone.0028013 (PMC3237429; doi:10.1371/journal.pone.0028013)
Supplement: Table S1 — Prevalence of infectious (parasitic/bacterial) and idiopathic conditions detected by histopathology and by pathogen screening (molecular and microscopic) in adult coho salmon sampled from several creeks and hatcheries in the Puget Sound region in 2003 and 2004. H = histopathology; PS = pathogen screening methods; − = analysis not performed. (DOC) [file pone.0028013.s003.doc]

Table S1. Prevalence of infectious (parasitic/bacterial) and idiopathic conditions detected by histopathology and by pathogen screening (molecular and microscopic) in adult coho salmon sampled from several creeks and hatcheries in the Puget Sound region in 2003 and 2004. H = histopathology; PS = pathogen screening methods; - = analysis not performed.

|  | Longfellow Creek | | Des Moines Creek | | Elliott Bay | | Issaquah Hatchery | | Wallace River Hatchery | |
| --- | --- | --- | --- | --- | --- | --- | --- | --- | --- | --- |
|  | H | PS | H | PS | H | PS | H | PS | H | PS |
| **GILL** |  |  |  |  |  |  |  |  |  |  |
| **N =** | 21 | 22 | 22 | 22 | 27 | 27 | 24 | 24 | 19 | 20 |
| *Ichthyophthirius* spp. (external ciliate) | 19% | - | 18% | - | 0% | - | 46% | - | 58% | - |
| *Loma* *salmonae* (microsporidan) | 24% | 55% | 9% | 4% | 7% | 15% | 13% | 4% | 5% | 5% |
| *Parvicapsula* *minibicornis* (myxozoan) | - | 32% | - | 0% | - | 7% | 0 | 79% | 0 | 100% |
| Respiratory epithelial hyperplasia | 33% | - | 18% | - | 59% | - | 63% | - | 47% | - |
| Microaneurysms | 5% | - | 0% | - | 7% | - | 29% | - | 0% | - |
| Monogenetic trematodes, external | 0% | - | 0% | - | 41% | - | 0% | - | 0% | - |
| **HEART** |  |  |  |  |  |  |  |  |  |  |
| **N =** | 21 | 0 | 22 | 0 | 26 | 0 | 24 | 0 | 20 | 0 |
| *Loma* *salmonae* (microsporidan) | 19% | - | 5% | - | 0% | - | 13% | - | 10% | - |
| *Nanophyetus* *salmincola* (larval trematodes) | 5% | - | 5% | - | 0% | - | 4% | - | 0% | - |
| **TRUNK KIDNEY** |  |  |  |  |  |  |  |  |  |  |
| **N =** | 21 | 21 | 21 | 14 | 27 | 27 | 23 | 24 | 20 | 20 |
| *Myxidium* sp. or *Parvicapsula* sp. (myxosporean) | 67% | - | 43% | - | 0% | - | 13% | - | 85% | - |
| *Parvicapsula* *minibicornis* (myxozoan) | - | 67% | - | 0% | - | 100% | - | 88% | - | 95% |
| *Nanophyetus* *salmincola* | 10% | 36% a | 19% | - | 19% | 48% | 0% | 0% | 0% | 0% |
| *Loma* *salmonae* | 0% | - | 0% | - | 0% | - | 4% | - | 0% | - |
| *Tetracapsula* *bryosalmonae* (myxozoan) | - | 10% | - | 0% | - | 7% | - | 0% | - | 40% |
| *Ceratomyxa* *shasta* (myxosporean) | 0% | - | 0% | - | 0% | - | 4% | - | 0% | - |
| *Renibacterium* *salmoninarum* (bacterium) | - | 52% | - | 100% | - | 22% | - | 33% | - | 30% |
| Tubular protein casts, nephrosis | 5% | - | 52% | - | 0% | - | 4% | - | 0% | - |
| **GASTROINTESTINAL TRACT (pyloric caeca, stomach, upper & lower intestine)** | | | | | | | | | | |
| **N =** | 21 | 14 | 22 | 0 | 27 | 27 | 24 | 24 | 19 | 20 |
| *Ceratomyxa shasta* | 5% | 0% | 0% | - | 0% | 18% | 75% | 4% | 0% | 0% |
| Cestodes, luminal | 33% | - | 45% | - | 30% | - | 13% | - | 32% | - |
| Trematodes, digeneans | 5% | - | 0% | - | 4% | - | 0% | - | 0% | - |
| Nematodes, mesenteric | 0% | - | 0% | - | 4% | - | 4% | - | 5% | - |
| Myxosporean sp. | 0% | - | 5% | - | 0% | - | 0% | - | 0% | - |
| **LIVER** |  |  |  |  |  |  |  |  |  |  |
| **N =** | 20 | 0 | 22 | 0 | 27 | 0 | 21 | 0 | 18 | 0 |
| Hydropic degenerative coagulative necrosis | 25%** | - | 36%*,** | - | 0% | - | 19% | - | 17% | - |
| Hepatocellular megalocytosis, nuclear pleomorphism | 15%*,** | - | 9%** | - | 0% | - | 0% | - | 6% | - |

* =significantly higher prevalence than at combined sites of Elliott Bay, Wallace River and Issaquah Creek hatcheries, by the Fisher’s Exact Test. ** =significantly higher prevalence at the combined sites of Longfellow and Des Moines Creeks than at the combined sites of Elliott Bay, Wallace River and Issaquah Creek hatcheries, by the Fisher’s Exact Test.

^a^ represents data for 14 fish (2003 only).
